# Serum Osmolality and Stroke Mortality in the ICU: A U-Shaped Risk Pattern and Its Clinical Implications

**DOI:** 10.3390/jcm14186406

**Published:** 2025-09-11

**Authors:** Ge Li, Wenshi Wei

**Affiliations:** Department of Neurology, Huadong Hospital Affiliated to Fudan University, 221 Yan’an West Road, Shanghai 200040, China; 24211280019@m.fudan.edu.cn

**Keywords:** serum osmolality, stroke, mortality, intensive care unit (ICU), prognosis

## Abstract

**Background:** Serum osmolality (SOSM) is a simple and objective tool for assessing hydration status and has been demonstrated in several studies to hold significant prognostic value in patients with cardiovascular and cerebrovascular diseases. This study aims to evaluate the association between SOSM and clinical outcomes in patients with stroke. **Methods:** This study evaluated the prognostic relevance of SOSM in stroke patients using data from the MIMIC-IV database. Eligible participants were divided into four quartile groups based on their SOSM: Q1, 277.62–296.30 mOsm/kg H_2_O; Q2, 296.31–301.60 mOsm/kg H_2_O; Q3, 301.61–307.74 mOsm/kg H_2_O; and Q4, 307.75–327.50 mOsm/kg H_2_O. This study used Cox proportional hazards regression, subgroup analysis, and restricted cubic spline analysis to examine the association between SOSM and mortality. **Results:** This study included 6005 stroke patients. The 30-day and 365-day mortality rates were 13.49% and 15.84%, respectively. After adjusting for relevant confounders, multivariate Cox regression analysis showed that higher SOSM was independently associated with an increased risk of 30-day death (HR: 1.83; 95% CI: 1.48–2.27; *p* < 0.001) and 365-day death (HR: 1.71; 95% CI: 1.41–2.08; *p* < 0.001). Analyses using restricted cubic splines (RCSs) and threshold effect modeling demonstrated a clear U-shaped relationship between SOSM and both short-term and long-term risk of death in stroke patients. Furthermore, subgroup analyses confirmed the stability of this association across diverse patient profiles. **Conclusions:** SOSM is independently associated with both 30-day and 365-day mortality in stroke patients. Our findings indicate that SOSM may be an effective indicator for stratifying high-risk patients who might benefit from targeted interventions, ultimately improving prognostic outcomes.

## 1. Introduction

Stroke is an acute neurological disorder characterized by an interruption in cerebral blood flow or intracerebral hemorrhage, leading to a sudden loss of brain function. The clinical manifestations of stroke are influenced by the location and degree of the insult, often resulting in significant neurological deficits such as hemiplegia, aphasia, and altered or impaired consciousness [1]. In the absence of timely and effective intervention, stroke is frequently associated with high rates of disability and mortality.

Recent epidemiological studies have reported a substantial increase in both the incidence and mortality rates of stroke globally [2]. This increasing trend emphasizes the growing public health challenge created by cerebrovascular diseases. Despite notable advancements in pharmacological treatments, endovascular therapy, and post-stroke rehabilitation, stroke remains a primary cause of disability and mortality, especially in patients admitted to intensive care units (ICUs) [3]. Consequently, the identification of reliable biomarkers for individualized risk assessment remains a critical priority in contemporary stroke research.

Serum osmolality (SOSM), defined as the osmotic pressure exerted by solute particles within plasma, represents a crucial physiological parameter essential for maintaining fluid balance and normal cellular function [4]. Its routine measurement in laboratory evaluations makes it a practical and objective tool for assessing hydration status across diverse patient populations [5]. In addition to its role in assessing fluid and sodium homeostasis, SOSM is employed to evaluate effective osmolality (tonicity) and the osmolal gap, which can aid in identifying pathological conditions such as toxic ingestions or metabolic disturbances [6]. Recent clinical guidance underscores that SOSM remains the gold-standard biomarker for identifying dehydration in clinical settings, despite some limitations in acute isotonic states [7]. Furthermore, SOSM has emerged as a prognostic biomarker in various clinical contexts. For instance, Gui et al. identified a J-shaped association between SOSM and risk of death among critically ill patients suffering from heart attack [8]. Similarly, Zhao et al. found that elevated SOSM (≥295 mOsm/kg H_2_O) was significantly linked to an increased risk of in-hospital death among patients suffering from intracerebral hemorrhage [9]. However, evidence regarding the prognostic significance of SOSM remains inconsistent. Liu et al. reported that elevated SOSM (>296 mOsm/kg) independently predicted 3-month mortality in patients with severe ischemic stroke, but this association was not sustained at 1 year (HR = 1.51, 95% CI: 0.84–2.72, *p* = 0.165) [10]. Likewise, in a cohort of respiratory failure patients, Çelik et al. found no significant link between SOSM and mortality [11]. Such divergent results suggest that the predictive value of SOSM may be context-dependent, reinforcing the need for further validation in larger and more heterogeneous cohorts.

Large-scale studies specifically examining the relationship between SOSM and mortality among patients with severe stroke continue to be insufficient. Consequently, this present study seeks to utilize the MIMIC-IV database to evaluate the prognostic relevance of SOSM in stroke patients, thereby advancing its potential clinical applications.

## 2. Materials

### 2.1. Study Population

Data for this retrospective research were obtained from the Medical Information Mart for Intensive Care IV (MIMIC-IV), version 3.1 [12]. The database contains detailed clinical data from ICU patients of Beth Israel Deaconess Medical Center, a Harvard Medical School teaching hospital [12].

**Figure 1:**
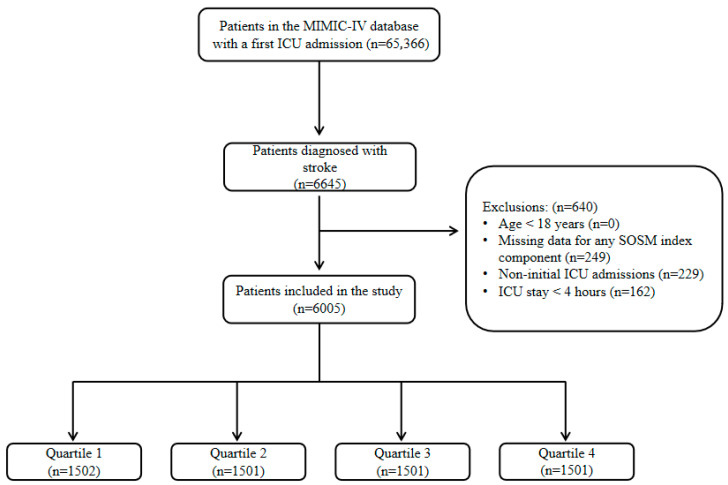
Flow diagram of patient selection and grouping. A total of 6005 stroke patients were included and stratified into SOSM quartiles.

Data extraction was conducted by a certified researcher (Ge Li) who successfully obtained authorized access in accordance with the database’s data use protocols. Stroke patients were identified using relevant ICD-9 and ICD-10 diagnostic codes documented during hospitalization. The study population included individuals admitted to the ICU between 2008 and 2019. We initially extracted ICU-admitted patients diagnosed with stroke from the MIMIC-IV database. To maintain data quality, we excluded patients based on the following criteria: (1) age less than 18 years, (2) missing essential laboratory values (serum sodium, potassium, glucose, and blood urea nitrogen (BUN)) within the first 24 h of ICU admission, and (3) non-initial ICU admissions or ICU stays lasting less than 4 h. Eligible patients were further stratified into quartiles according to SOSM values: Q1, 277.62–296.30 mOsm/kg H_2_O; Q2, 296.31–301.60 mOsm/kg H_2_O; Q3, 301.61–307.74 mOsm/kg H_2_O; and Q4, 307.75–327.50 mOsm/kg H_2_O. A detailed overview of the patient selection process is depicted in Figure 1.

### 2.2. Data Extraction

Structured data were extracted using SQL queries, enabling efficient and reproducible retrieval of relevant patient information from the MIMIC-IV (version 3.1) database. Extracted variables were systematically categorized into five major groups to facilitate comprehensive analysis: (1) Demographics: age, sex, race, and weight. (2) Comorbidities: Chronic and acute conditions known to affect prognosis and treatment response were identified, including atrial fibrillation, diabetes mellitus, heart failure, chronic obstructive pulmonary disease (COPD), acute kidney injury (AKI), hypertension, pneumonia, and ischemic heart disease. (3) Laboratory indicators: Routine laboratory measurements reflecting physiological status and organ function were extracted, including white blood cell (WBC) count, red blood cell (RBC) count, platelet count, potassium, BUN, hemoglobin, serum creatinine (Scr), prothrombin time (PT), partial thromboplastin time (PTT), and glucose. All laboratory values were obtained within the first 24 h after ICU admission, and if multiple results were available within this period, the average value was calculated and used for analysis. (4) Illness severity scores: Established scoring systems were used to evaluate disease severity upon ICU admission, including the Glasgow Coma Scale (GCS), Oxford Acute Severity of Illness Score (OASIS), Acute Physiology Score III (APS III), Systemic Inflammatory Response Syndrome (SIRS) criteria, Simplified Acute Physiology Score II (SAPS II), and Sequential Organ Failure Assessment (SOFA) score. (5) Medications: antilipemic, antiplatelet agents, and thrombolytic therapy. SOSM was calculated using the following formula [13]:

SOSM = 2 × [Na^+^(mmol/L) + K^+^(mmol/L)] + glucose(mg/dL)/18 + BUN (mg/dL)/2.8

### 2.3. Outcomes

The primary outcome of this study was 30-day mortality, serving as a measure of short-term survival after hospital admission. The secondary outcome was 365-day mortality, providing insight into long-term survival following hospitalization.

### 2.4. Statistical Analysis

Statistical analyses were conducted using R software (version 4.2.3). Continuous variables were reported as median with interquartile ranges (IQR) and compared across the four SOSM quartile groups using the one-way analysis of variance (ANOVA) or Kruskal–Wallis test. Categorical variables were expressed as counts with percentages and compared using the Chi-square test or Fisher’s exact test, when appropriate. Survival curves were generated with the Kaplan–Meier method and compared using the log-rank test. Univariate and multivariate Cox proportional hazards regression analyses were performed to estimate HRs with 95% CIs for both 30-day and 365-day mortality. To assess potential nonlinear associations, restricted cubic spline (RCS) functions with three knots were applied. Subgroup analyses were conducted to test the robustness of the findings. Statistical significance was defined as a two-tailed *p* < 0.050.

## 3. Results

### 3.1. Study Population

This study comprised 6005 stroke patients admitted to the ICU. The median age was 76.90 years (IQR: 64.70 to 90.00), and 53.39% were men. The 30-day and 365-day mortality rates were 13.49% and 15.84%, respectively. Patients were categorized into four groups (Q1 to Q4) according to SOSM quartiles. We compared demographic characteristics, clinical severity scores, laboratory findings, comorbidities, and both short- and long-term mortality across the four groups. P values in Table 1 represent overall comparisons among the four SOSM quartiles. Normally distributed continuous variables were tested using one-way ANOVA, non-normally distributed continuous variables were tested using the Kruskal–Wallis test, and categorical variables were tested using the chi-square test or Fisher’s exact test, as appropriate.

As presented in Table 1, patients in the Q4 group had the highest 30-day and 365-day mortality rates (22.65% and 26.25%, respectively), significantly exceeding those in the other groups. Furthermore, the prevalence of comorbidities, including heart failure, diabetes, AKI, COPD, atrial fibrillation, pneumonia, and ischemic heart disease, was also highest in the Q4 group. Severity scores such as SOFA, APS III, SAPS II, and OASIS were likewise elevated in Q4, indicating more severe clinical conditions.

### 3.2. Primary Outcomes

**Figure 2:**
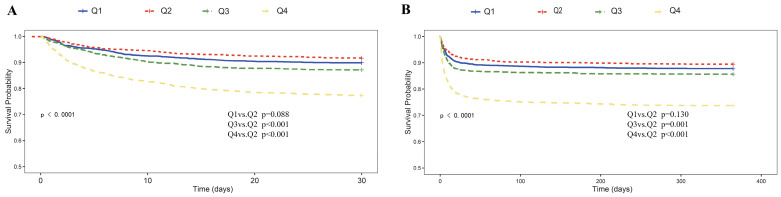
Kaplan–Meier survival analysis curves for 30-day (**A**) and 365-day (**B**) all-cause mortality in patients with stroke.

Kaplan–Meier (KM) survival analysis was constructed to assess survival differences among the four groups. Survival distributions were compared using the log-rank test, and the *p* values reflect overall differences across the four SOSM quartiles. Significant differences were observed in both short-term and long-term survival across groups (*p* < 0.001). As shown in Figure 2, patients in the highest SOSM quartile (Q4) had the highest 30-day and 365-day mortality risks, while those in the Q2 group had the lowest. Notably, while pairwise comparisons indicated that Q3 and Q4 had significantly worse outcomes compared with Q2, the difference between Q1 and Q2 did not reach statistical significance. These findings suggest that elevated SOSM (Q3 and Q4) was consistently associated with excess mortality, whereas reduced SOSM (Q1) demonstrated a similar trend but did not achieve statistical significance in the KM analysis.

**Figure 3:**
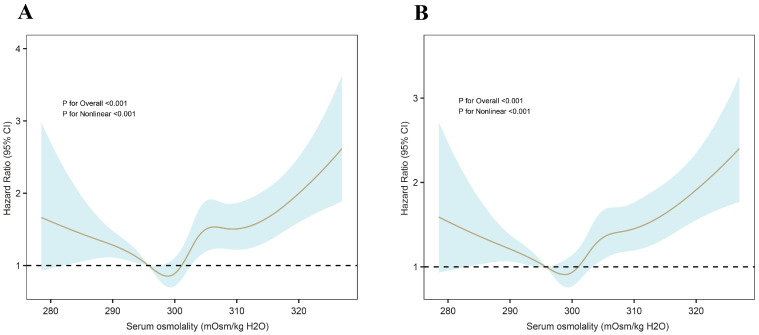
RCS analysis illustrating the hazard ratios for SOSM in relation to (**A**) 30-day and (**B**) 365-day all-cause mortality.

Restricted cubic spline (RCS) analysis revealed that SOSM exhibits a U-shaped relationship with the risk of both short-term and long-term mortality among patients with stroke. The nonlinearity of the association was formally tested using a likelihood ratio test, with *p* < 0.001 indicating a significant departure from linearity. As shown in Figure 3, both hypo-osmolality and hyper-osmolality were associated with increased mortality. Threshold analysis further identified inflection points for SOSM: 297.24 mOsm/kg H_2_O for 30-day mortality and 297.2 mOsm/kg H_2_O for 365-day mortality.

Threshold effect analysis was performed using a two-piece Cox proportional hazard regression model. As shown in Table 2, when SOSM exceeded 297.24 mOsm/kg H_2_O, each 1 mOsm/kg H_2_O increase corresponded to a 3.4% (95% CI: 2.4–4.4%) increase in risk of 30-day mortality. Conversely, when SOSM was below this threshold, each 1 mOsm/kg H_2_O decrease was associated with a 3.2% (95% CI: 0.9–5.4%) increase in risk. Similarly, for 365-day mortality, each 1 mOsm/kg H_2_O increase above 297.20 mOsm/kg H_2_O corresponded to a 3.2% (95% CI: 2.3–4.1%) increase in risk, while each 1 mOsm/kg H_2_O decrease below 297.20 mOsm/kg H_2_O was associated with a 2.9% (95% CI: 0.8–5.0%) increase in risk.

As shown in Table S1 (Supplementary Materials), univariate analysis identified 22 variables significantly associated with the risk of death in stroke patients (*p* < 0.05). These included age, weight, race, AKI, heart failure, hypertension, pneumonia, WBC, RBC, platelet count, hemoglobin, Scr, BUN, PT, PTT, glucose, potassium, APS III, OASIS, SAPS II, SIRS, SOFA score, and SOSM.

These significant variables were subsequently incorporated into multivariable Cox regression models (Table 3). Model 1 was unadjusted, Model 2 was adjusted for age, race, sex, and weight, and Model 3 was further adjusted for AKI, hypertension, heart failure, pneumonia, WBC, RBC, platelet count, hemoglobin, Scr, BUN, PT, PTT, glucose, potassium, APS III, OASIS, SAPS II, SIRS, and SOFA score. When SOSM was treated as a continuous variable, it remained an independent risk factor for mortality in all three models: Model 1: HR = 1.05 (95% CI: 1.04–1.05), *p* < 0.001; Model 2: HR = 1.04 (95% CI: 1.04–1.05), *p* < 0.001; Model 3: HR = 1.03 (95% CI: 1.02–1.05), *p* < 0.001. When SOSM was categorized into quartiles, using Q2 as the reference group, short-term mortality risk was significantly higher in Q3 and Q4: Model 1: Q3 HR = 1.59 (95% CI: 1.27–1.99), *p* < 0.001; Q4 HR = 2.98 (95% CI: 2.43–3.66), *p* < 0.001; Model 2: Q3 HR = 1.54 (95% CI: 1.23–1.93), *p* < 0.001; Q4 HR = 2.79 (95% CI: 2.27–3.43), *p* < 0.001; Model 3: Q1 HR = 1.30 (95% CI: 1.03–1.65), *p* = 0.031; Q3 HR = 1.54 (95% CI: 1.23–1.93), *p* < 0.001; Q4 HR = 1.83 (95% CI: 1.48–2.27), *p* < 0.001.

Notably, the elevated risk in Q1 emerged only after full adjustment, indicating that the adverse impact of reduced SOSM may be partly masked in unadjusted survival comparisons. A similar trend was observed in the association between SOSM and long-term mortality among stroke patients. Taken together, these findings underscore that both elevated and reduced SOSM are independently associated with increased mortality risk, with the lowest risk consistently observed in the mid-range (Q2). All hazard ratios were derived from Cox proportional hazards regression, with p values reflecting the statistical significance of differences in mortality risk across SOSM quartiles or per-unit changes in SOSM.

**Figure 4:**
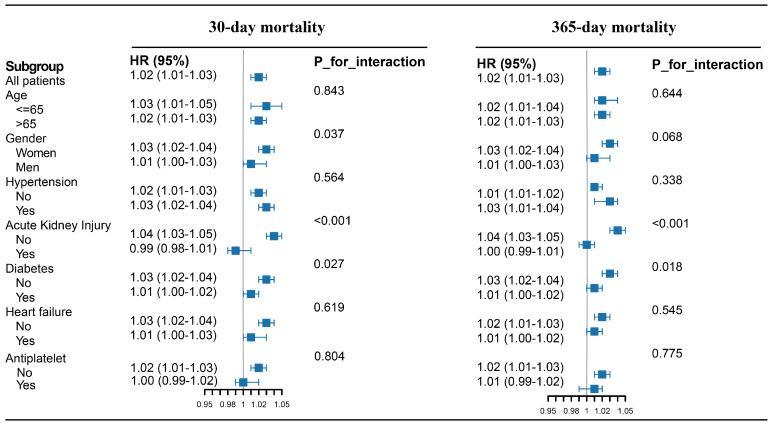
Forest plots of hazard ratios for the 30- and 365-day mortality in different subgroups.

After controlling for relevant confounders, we further evaluated whether the predictive value of SOSM for short- and long-term mortality varied across different subgroups of stroke patients, including age, sex, hypertension, AKI, diabetes, heart failure, and use of antiplatelet agents (Figure 4). The *p* values shown in Figure 4 reflect the statistical significance of interaction tests, indicating whether the association between SOSM and mortality risk differed significantly across subgroups.

In the overall cohort (n = 6005), each 1 mOsm/kg H_2_O increase in SOSM corresponded to a 2% increase in mortality risk (HR = 1.02; 95% CI: 1.01–1.03; *p* < 0.001). Subgroup analyses defined by age, hypertension, heart failure, and antiplatelet agent use showed consistent effects of SOSM (all interaction *p* > 0.050), with HRs ranging from 1.00 to 1.03.

However, significant interactions were observed in the sex and diabetes subgroups. Compared with men (HR = 1.01; 95% CI: 1.00–1.03), women showed a greater mortality risk associated with SOSM (HR = 1.03; 95% CI: 1.02–1.04). Similarly, SOSM remained a stronger association with mortality in non-diabetic patients, with each 1 mOsm/kg H_2_O increase corresponding to a 3% increase in risk (95% CI: 1.02–1.04), while the association was weaker and not statistically significant among diabetic patients (HR = 1.01; 95% CI: 1.00–1.02).

Similar trends were observed in the subgroup analysis for long-term mortality. In the full cohort, each 1 mOsm/kg H_2_O increase in SOSM corresponded to a 2% increase in long-term mortality risk (HR = 1.02; 95% CI: 1.01–1.03; *p* < 0.001). Unlike the findings for short-term mortality, the effect of SOSM on long-term outcomes was consistent across sex subgroups (interaction *p* = 0.068). Nonetheless, SOSM remained a stronger association with long-term mortality in non-diabetic patients.

## 4. Discussion

This study aimed to evaluate the relationship between SOSM and prognosis in ICU-admitted stroke patients. The analysis identified a clear U-shaped relationship between SOSM and both 30- and 365-day mortality, indicating that both high and low SOSM levels were linked to a higher risk of mortality. Threshold effect analysis identified inflection points at 297.24 mOsm/kg H_2_O for 30-day mortality and 297.20 mOsm/kg H_2_O for 365-day mortality. After adjusting for potential confounders, these associations remained statistically significant. Our findings indicate that SOSM may be a valuable prognostic marker to guide clinical management in critically ill stroke patients.

SOSM reflects the total osmotic pressure exerted by all dissolved solutes in the serum, primarily electrolytes and small organic molecules [4]. Sodium ions (Na^+^) and their accompanying anions account for approximately 95% of effective osmolality, while glucose and BUN also make significant contributions [14]. SOSM plays a vital role in maintaining fluid balance between intracellular and extracellular compartments, as well as preserving normal cellular structure and function [15]. Water molecules naturally move from areas of lower to higher SOSM in an effort to equalize solute concentrations. The normal reference range for SOSM is typically 280 to 300 mOsm/kg H_2_O [16].

Several retrospective clinical studies have reported associations between SOSM and patient outcomes in a variety of disease states. For example, Hakkl et al. found that in patients with heart failure, elevated plasma osmolality was an independent prognostic indicator of all-cause mortality, yielding a HR of 3.54 (95% CI: 1.72–7.30) when osmolality exceeded 295 mOsm/kg H_2_O [17]. Similarly, Hu et al. analyzed data from 9547 adults with diabetes in the U.S. Veterans Affairs database and identified a significant U-shaped nonlinear association between SOSM and cardiovascular mortality, with an increased risk observed when SOSM was either above or below 297 mOsm/kg H_2_O [18].

Among critically ill individuals with myocardial infarction (MI), Gui et al. observed a J-shaped association between SOSM and all-cause mortality [8]. Mortality risk increased sharply when SOSM fell below 280 mOsm/kg H_2_O, remained lowest in the 280–295 mOsm/kg H_2_O range, and rose again when SOSM exceeded 295 mOsm/kg H_2_O. Our findings are consistent with these studies, identifying an inflection point of 297.24 mOsm/kg H_2_O for short-term mortality. Despite differences in underlying disease conditions and mechanisms, this threshold appears to be relatively consistent across populations and may have broad prognostic relevance.

Additionally, Anne et al. observed that nearly half of stroke patients presented with hyper-osmolality upon admission, regardless of stroke subtype, and that this condition was associated with worse clinical outcomes [19]. These findings underscore the importance of SOSM monitoring. By utilizing a large ICU database, our study adds to the accumulating scientific evidence supporting the prognostic significance of SOSM in critically ill stroke patients and strengthens the credibility of the observed associations.

The exact mechanisms linking SOSM to cerebrovascular outcomes remain incompletely understood. Elevated SOSM often reflects a hyper-osmolar or dehydrated state, which may lead to hemoconcentration and increased blood viscosity [9,20]. These hemodynamic alterations can reduce cerebral perfusion, particularly in ischemic or vulnerable brain regions, thereby aggravating focal ischemic injury, enlarging infarct size, and increasing the risk of stroke recurrence [19,21].

Moreover, hyper-osmolality may induce a hypercoagulable state that increases the likelihood of thrombosis and further compromises cerebral blood flow [22]. It can also activate multiple neuroendocrine pathways, including the renin–angiotensin–aldosterone system (RAAS) and the secretion of antidiuretic hormone [23]. Although these responses may offer temporary physiological compensation, their excessive activation may impair renal perfusion and contribute to the AKI [24].

In critically ill stroke patients, AKI can alter the pharmacokinetics and clearance of important medications, such as antihypertensive and neuroprotective agents. These alterations may reduce therapeutic efficacy or increase the risk of toxicity [25]. AKI is also associated with electrolyte disturbances, especially hypernatremia and hyperglycemia [6]. Donghua Mi et al. reported that in patients with acute ischemic stroke, admission blood glucose levels exceeding 7.8 mmol/L were associated with a progressively higher mortality risk, and tighter glycemic control was linked to improved prognosis [26].

In addition, hyper-osmolality-induced neuronal dehydration may disrupt membrane integrity and impair neurological function. Dehydration also increases the viscosity of respiratory secretions and weakens mucociliary clearance. This is particularly concerning in stroke patients with dysphagia, who face an elevated risk of aspiration [27]. The development of secondary aspiration pneumonia complicates clinical management, prolongs hospitalization, and is strongly associated with poor outcomes [28].

Although much of the literature has focused on the harmful effects of hyper-osmolality, the pathophysiological consequences of hypo-osmolality also deserve attention. Reduced SOSM typically indicates dilutional hyponatremia or impaired renal clearance, both of which promote water influx into brain cells, resulting in cytotoxic cerebral edema and raised intracranial pressure [29,30]. In addition, hyponatremia can disrupt neuronal excitability, thereby increasing the risk of seizures and accelerating neurological decline [31,32]. These mechanisms offer a plausible explanation for the higher mortality observed in patients with SOSM values below the identified threshold.

Collectively, these multifactorial pathophysiological processes may explain the observed association between both elevated and decreased SOSM and adverse outcomes in stroke patients. Additional research is needed to clarify the potential mechanisms and to evaluate whether interventions targeting SOSM could improve clinical prognosis.

Our RCS analysis revealed a distinct U-shaped association between SOSM and both short- and long-term mortality in critically ill stroke patients, indicating that departures from the midrange are linked with increased risk. Threshold modeling identified inflection points near 297.2 mOsm/kg H_2_O, marking the zone of minimal mortality. From a clinical perspective, values below this range may reflect conditions such as dilutional hyponatremia, fluid overload, or impaired renal clearance, each of which can aggravate cerebral edema and worsen neurological outcomes [33]. In contrast, higher SOSM values suggest dehydration or hypernatremia, which may lead to hemoconcentration, reduced cerebral perfusion, and further ischemic damage [34]. These findings highlight the potential importance of maintaining SOSM within an optimal interval around 297.2 mOsm/kg H_2_O to support better prognostic outcomes in stroke care. Our study demonstrated that SOSM remained significantly associated with stroke prognosis even after adjusting for multiple confounders (HR = 1.03, 95% CI: 1.02–1.05, *p* < 0.001). Subgroup analyses confirmed the stability of this association across various patient characteristics, including age, sex, and comorbidities. Notably, the predictive value of SOSM was greater in non-diabetic patients. These results suggest that SOSM, as a simple and widely accessible clinical parameter, may serve as a practical tool for early risk assessment in critically ill patients with stroke. The early recognition of high-risk individuals may enable timely intervention and potentially improve clinical outcomes. In addition, the monitoring and regulation of SOSM may offer a valuable adjunctive approach to current treatment strategies.

Although serum osmolality is not disease-specific and may in part reflect overall health status, it remains a strong prognostic indicator in critically ill stroke patients. The ability of a single admission value to predict long-term outcomes likely reflects its capacity to integrate multiple physiological domains, including hydration balance, renal clearance, and systemic resilience. This broad sensitivity, while limiting specificity, underscores the practical utility of SOSM as a low-cost and readily obtainable marker for early risk stratification. Future research with serial measurements will be important to determine whether SOSM primarily captures stroke-related pathophysiology or more general indicators of physiological reserve.

Nonetheless, there are several notable limitations that must be recognized. First, the retrospective design of the MIMIC-IV database limits the ability to establish causal relationships. Second, SOSM was measured only within the first 24 h after ICU admission. The absence of dynamic monitoring hindered the assessment of longitudinal changes and their prognostic significance. Future studies should incorporate repeated or continuous measurements of SOSM during hospitalization and apply longitudinal analytic approaches, such as mixed-effects models or trajectory-based methods, to better characterize temporal patterns. Third, data on certain important confounding variables, such as the type and volume of fluid resuscitation and specific interventions aimed at osmolality regulation, were not available. Fourth, detailed information regarding stroke subtypes and neuroimaging findings was limited, which may have impacted the accuracy of stroke severity classification. Finally, potential selection bias inherent to the MIMIC-IV database must be considered, as the data were derived from a single tertiary center in the United States. This may limit generalizability due to geographic, demographic, and severity-related differences, underscoring the need for external validation in multicenter and international cohorts.

Future studies should aim to assess these findings in prospective, multicenter settings. Additional research is needed to explore the prognostic value of dynamic changes in SOSM, from pre-hospital admission through ICU care and discharge [35]. Moreover, integrating imaging results, biomarker data, and inflammatory indicators into composite prognostic models may enhance predictive accuracy. Importantly, interventional studies are required to determine whether correction of hyper-osmolality can improve outcomes in stroke patients and serve as a novel therapeutic target.

## 5. Conclusions

In conclusion, SOSM showed a clear U-shaped association with both short- and long-term mortality in ICU stroke patients, and it remained an independent predictor even after adjustment for confounding factors. Early monitoring within the first 24 h of admission may help identify those at highest risk, allowing for timely fluid and electrolyte management. These findings suggest that SOSM could serve as a practical and cost-effective tool for risk stratification in critical care settings.

## Figures and Tables

**Table 1:** Characteristics and outcomes of patients with stroke categorized by serum osmolality.

Variable	Overall (N = 6005)	Q1 (N = 1502)	Q2 (N = 1501)	Q3 (N = 1501)	Q4 (N = 1501)	*p*-Value
**Demographic characteristics**						
Age (years)	74.00 (63.00–83.00)	72.00 (61.00–82.00)	72.00 (62.00–82.00)	74.00 (64.00–82.00)	77.00 (67.00–85.00)	<0.001
Weight (kg)	76.90 (64.70–90.00)	75.30 (63.00–87.85)	77.20 (65.50–89.50)	77.20 (65.50–91.25)	77.10 (65.00–92.20)	<0.001
Sex: men (n, %)	3206.00 (53.39%)	777.00 (51.73%)	826.00 (55.03%)	792.00 (52.76%)	811.00 (54.03%)	<0.001
RACE (n, %)						<0.001
BLACK	632.00 (10.52%)	136.00 (9.05%)	133.00 (8.86%)	150.00 (9.99%)	213.00 (14.19%)	
WHITE	4247.00 (70.72%)	1088.00 (72.44%)	1089.00 (72.55%)	1069.00 (71.22%)	1001.00 (66.69%)	
OTHER	1126.00 (18.75%)	278.00 (18.51%)	279.00 (18.59%)	282.00 (18.79%)	287.00 (19.12%)	
**Clinical score**						
GCS (score)	15.00 (13.00–15.00)	14.00 (13.00–15.00)	14.00 (13.00–15.00)	15.00 (13.00–15.00)	14.00 (13.00–15.00)	0.091
SOFA (score)	4.00 (2.00–6.00)	3.00 (2.00–5.00)	3.00 (2.00–5.00)	3.00 (2.00–5.00)	5.00 (3.00–7.00)	<0.001
APSIII (score)	39.00 (29.00–51.00)	34.00 (26.00–46.00)	35.00 (27.00–45.00)	38.00 (30.00–49.00)	48.00 (38.00–63.00)	<0.001
SAPSII (score)	35.00 (28.00–43.00)	32.00 (25.00–40.00)	32.00 (26.00–40.00)	35.00 (28.00–42.00)	40.00 (33.00–50.00)	<0.001
OASIS (score)	30.00 (25.00–36.00)	29.00 (24.00–35.00)	29.00 (24.00–35.00)	30.00 (25.00–36.00)	33.00 (27.00–39.00)	<0.001
**Commorbidities**						
Heart failure (n, %)	1851.00 (30.82%)	349.00 (23.24%)	375.00 (24.98%)	477.00 (31.78%)	650.00 (43.30%)	<0.001
Diabetes (n, %)	2082.00 (34.67%)	382.00 (25.43%)	436.00 (29.05%)	554.00 (36.91%)	710.00 (47.30%)	<0.001
COPD (n, %)	931.00 (15.50%)	229.00 (15.25%)	215.00 (14.32%)	232.00 (15.46%)	255.00 (16.99%)	0.241
Arterial fibrillation (n, %)	1131.00 (18.83%)	282.00 (18.77%)	250.00 (16.66%)	271.00 (18.05%)	328.00 (21.85%)	0.003
Hypertension (n, %)	3078.00 (51.26%)	855.00 (56.92%)	873.00 (58.16%)	784.00 (52.23%)	566.00 (37.71%)	<0.001
Acute Kidney Injury (n, %)	1674.00 (27.88%)	250.00 (16.64%)	278.00 (18.52%)	406.00 (27.05%)	740.00 (49.30%)	<0.001
Pneumonia (n, %)	1141.00 (19.00%)	253.00 (16.84%)	245.00 (16.32%)	244.00 (16.26%)	399.00 (26.58%)	<0.001
Ischemic Heart Disease (n, %)	2499.00 (41.62%)	573.00 (38.15%)	599.00 (39.91%)	616.00 (41.04%)	711.00 (47.37%)	<0.001
**Laboratory tests**						
WBC (K/μL)	10.40 (7.70–13.78)	10.32 (7.75–13.48)	10.20 (7.60–13.58)	10.30 (7.60–13.54)	10.80 (7.85–14.70)	0.004
RBC (m/μL)	3.65 (3.17–4.15)	3.61 (3.18–4.09)	3.78 (3.29–4.23)	3.74 (3.25–4.23)	3.46 (3.00–4.00)	<0.001
Platelet (K/μL)	193.50 (148.00–251.00)	198.67 (149.00–264.00)	197.00 (150.00–250.00)	190.80 (150.00–244.75)	190.00 (142.00–245.33)	<0.001
Hemoglobin (g/dL)	10.95 (9.47–12.45)	10.95 (9.50–12.40)	11.35 (9.87–12.70)	11.20 (9.70–12.60)	10.30 (8.85–11.93)	<0.001
Scr (mg/dL)	1.00 (0.75–1.40)	0.80 (0.65–1.00)	0.90 (0.70–1.20)	1.00 (0.80–1.30)	1.43 (1.00–2.24)	<0.001
BUN (mg/dL)	19.00 (14.00–29.25)	14.00 (10.67–19.00)	16.67 (13.00–22.50)	20.00 (15.50–27.67)	34.33 (23.00–49.67)	<0.001
PT (s)	14.00 (12.40–15.93)	13.85 (12.30–15.93)	13.80 (12.30–15.93)	13.70 (12.25–15.93)	14.60 (12.75–16.78)	<0.001
PTT (s)	31.80 (27.55–37.24)	31.90 (27.70–37.24)	31.37 (27.60–37.24)	31.30 (27.20–37.24)	32.75 (27.70–38.40)	0.002
Glucose (mg/dL)	127.00 (105.67–157.00)	116.00 (100.50–137.33)	122.00 (104.00–147.67)	129.50 (108.00–161.00)	145.00 (115.00–191.00)	<0.001
Sodium (mmol/L)	139.00 (136.67–141.00)	136.00 (134.00–137.00)	139.00 (137.20–140.00)	140.50 (138.80–142.00)	142.00 (139.50–144.00)	<0.001
Potassium (mmol/L)	4.10 (3.77–4.45)	4.03 (3.70–4.35)	4.05 (3.77–4.37)	4.10 (3.80–4.40)	4.20 (3.80–4.65)	<0.001
**Medications**						
Antiplatelet (n, %)	3419.00 (56.94%)	857.00 (57.06%)	889.00 (59.23%)	848.00 (56.50%)	825.00 (54.96%)	0.126
Statin (n, %)	1306.00 (21.75%)	319.00 (21.24%)	349.00 (23.25%)	320.00 (21.32%)	318.00 (21.19%)	0.447
IV-tPA (n, %)	298.00 (4.96%)	84.00 (5.59%)	76.00 (5.06%)	58.00 (3.86%)	80.00 (5.33%)	0.135
**Clinical outcomes**						
30-day mortality (n, %)	810.00 (13.49%)	152.00 (10.12%)	125.00 (8.33%)	193.00 (12.86%)	340.00 (22.65%)	<0.001
365-day mortality (n, %)	951.00 (15.84%)	184.00 (12.25%)	158.00 (10.53%)	215.00 (14.32%)	394.00 (26.25%)	<0.001
ICU stay (day)	2.04 (1.14–4.02)	1.94 (1.13–3.84)	1.99 (1.12–3.59)	2.02 (1.10–4.08)	2.23 (1.24–4.95)	<0.001
Hospital stay (day)	7.11 (4.04–12.65)	7.33 (4.27–12.46)	6.72 (3.97–11.89)	6.90 (3.96–11.95)	7.76 (4.03–13.95)	<0.001

Serum osmolality values: Q1, 277.62–296.30 mOsm/kg H_2_O; Q2, 296.31–301.60 mOsm/kg H_2_O; Q3, 301.61–307.74 mOsm/kg H_2_O; and Q4, 307.75–327.50 mOsm/kg H_2_O. Abbreviations: GCS, Glasgow Coma Scale; SOFA, Sequential Organ Failure Assessment; APSIII, Acute Physiology Score III; SAPSII, Simplified Acute Physiology Score II; OASIS, Oxford Acute Severity of Illness Score; COPD, Chronic Obstructive Pulmonary Disease; WBC, white blood cell count; RBC, red blood cell count; Scr, serum creatinine; BUN, blood urea nitrogen; PT, prothrombin time; PTT, partial thromboplastin time; tPA, tissue plasminogen activator; ICU, intensive care unit.

**Table 2:** Threshold effect analysis.

Outcomes	30-Day Mortality		365-Day Mortality	
	HR (95%CI)	*p*-Value	HR (95%CI)	*p*-Value
Threshold (W)	297.244		297.201	
<W	0.968 (0.946–0.991)	0.006	0.971 (0.950–0.992)	0.007
>W	1.034 (1.024–1.044)	<0.001	1.032 (1.023–1.041)	<0.001
Log-likelihood ratio test		<0.001		<0.001

**Table 3:** SOSM and mortality: Cox regression results.

Categories	Model 1			Model 2			Model 3		
	HR (95%CI)	*p*-Value	P for Trend	HR (95%CI)	*p*-Value	P for Trend	HR (95%CI)	*p*-Value	P for Trend
30-day mortality									
Continuous variable	1.05 (1.04–1.05)	<0.001		1.04 (1.04–1.05)	<0.001		1.03 (1.02–1.05)	<0.001	
per unit									
Quartile			<0.001			<0.001			<0.001
Q1 (N = 1502)	1.23 (0.97–1.56)	0.088		1.23 (0.97–1.56)	0.09		1.30 (1.03–1.65)	0.031	
Q2 (N = 1501)	Ref								
Q3 (N = 1501)	1.59 (1.27–1.99)	<0.001		1.54 (1.23–1.93)	<0.001		1.54 (1.23–1.93)	<0.001	
Q4 (N = 1501)	2.98 (2.43–3.66)	<0.001		2.79 (2.27–3.43)	<0.001		1.83 (1.48–2.27)	<0.001	
365-day mortality									
Continuous variable	1.05 (1.04–1.05)	<0.001		1.04 (1.03–1.05)	<0.001		1.02 (1.01–1.03)	<0.001	
per unit									
Quartile			<0.001			<0.001			<0.001
Q1 (N = 1502)	1.18 (0.95–1.46)	0.13		1.17 (0.95–1.45)	0.142		1.22 (0.99–1.51)	0.068	
Q2 (N = 1501)	Ref								
Q3 (N = 1501)	1.40 (1.14–1.72)	0.001		1.37 (1.11–1.68)	0.003		1.35 (1.10–1.66)	0.004	
Q4 (N = 1501)	2.77 (2.30–3.33)	<0.001		2.60 (2.158–3.13)	<0.001		1.71 (1.41–2.08)	<0.001	

Serum osmolality values: Q1, 277.62–296.30 mOsm/kg H_2_O; Q2, 296.31–301.60 mOsm/kg H_2_O; Q3, 301.61–307.74 mOsm/kg H_2_O; and Q4, 307.75–327.50 mOsm/kg H_2_O. Model 1: unadjusted. Model 2: adjusted for age, sex, race, and weight. Model 3: adjusted for age, sex, race, and weight, AKI, heart failure, hypertension, pneumonia, WBC, RBC, platelet count, hemoglobin, Scr, BUN, PT, PTT, glucose, potassium, APS III, OASIS, SAPS II, SIRS, and SOFA score.

## Data Availability

The data used in this study are freely available from the MIMIC-IV database (version 3.1), available online https://physionet.org/content/mimiciv/3.1/ accessed on 5 April 2025. The raw datasets and analysis code supporting the findings of this study are available from the corresponding author upon reasonable request.

## Supplementary Materials

The following supporting information can be downloaded at https://www.mdpi.com/article/10.3390/jcm14186406/s1, Table S1: Characteristics of stroke patients significantly associated with the risk of death.
