# Supplementary material for: Serum Osmolality and Stroke Mortality in the ICU: A U-Shaped Risk Pattern and Its Clinical Implications
Source: J Clin Med. 2025 Sep 11;14(18):6406. doi: 10.3390/jcm14186406 (PMC12470425; doi:10.3390/jcm14186406)
Supplement: Supplementary file 1 [file jcm-14-06406-s001.zip › jcm-3811141-supplementary.pdf]

Table S1. Characteristics of stroke patients significantly associated with the risk of death.

Characteristics	Hazard Ratio(HR)	lower 95	upper 95	Pvalue
age	1.027	1.021	1.033	<0.001
gender: male	0.873	0.761	1.002	0.053
weight	0.989	0.986	0.993	<0.001
race				
OTHER	1.729	1.339	2.233	<0.001
WHITE	0.954	0.752	1.209	0.695
AKI	1.867	1.623	2.147	<0.001
Diabetes	1.053	0.912	1.216	0.48
Heart failure	1.3	1.126	1.5	<0.001
Hypertension	0.861	0.75	0.988	0.033
Ischemic Heart Disease	1.058	0.921	1.216	0.426
Pneumonia	1.982	1.708	2.3	<0.001
Arterial fibrillation	1.1	0.927	1.305	0.275
WBC, K/uL	1.024	1.019	1.029	<0.001
RBC, m/uL	0.862	0.779	0.955	0.004
Platelet, K/uL	1	0.999	1	0.498
Hemoglobin, g/dL	0.952	0.92	0.985	0.005
Scr, mg/dl	1.147	1.109	1.187	<0.001
BUN, mg/dl	1.02	1.017	1.024	<0.001
pt, sec	1.028	1.023	1.033	<0.001
ptt, sec	1.009	1.006	1.012	<0.001
glucose, mg/dl	1.006	1.005	1.007	<0.001
Sodium, mmol/L	0.994	0.976	1.013	0.552
Potassium, mmol/L	1.326	1.181	1.488	<0.001
apsiii	1.036	1.034	1.039	<0.001
oasis	1.103	1.095	1.111	<0.001
sapsii	1.061	1.056	1.065	<0.001
sirs	1.762	1.633	1.901	<0.001
sofa	1.219	1.198	1.24	<0.001
osmolality	1.046	1.039	1.054	<0.001
